# Total body water estimations in healthy men and women using bioimpedance spectroscopy: a deuterium oxide comparison

**DOI:** 10.1186/1743-7075-5-7

**Published:** 2008-03-19

**Authors:** Jordan R Moon, Sarah E Tobkin, Michael D Roberts, Vincent J Dalbo, Chad M Kerksick, Michael G Bemben, Joel T Cramer, Jeffrey R Stout

**Affiliations:** 1 Metabolic and body composition laboratories, Department of Health and Exercise Science, University of Oklahoma, Norman, OK, USA; 2 Biophysics laboratory, Department of Health and Exercise Science, University of Oklahoma, Norman, OK, USA; 3 Neuromuscular laboratory, Department of Health and Exercise Science, University of Oklahoma, Norman, OK, USA; 4 Applied biochemistry and molecular physiology laboratory, Department of Health and Exercise Science, University of Oklahoma, Norman, OK, USA

## Abstract

**Background:**

Total body water (TBW) estimations have been used to estimate body composition, particularly fat-free mass, to aid in nutritional interventions, and to monitor hydration status. In the past, bioimpedance spectroscopy (BIS) devices have been used to estimate TBW. Previous investigations have examined the validity of the XiTRON 4000B (XiTRON Technologies) BIS device for estimating TBW. Recently, a new BIS device (Imp™ SFB7) has become available, claiming greater precision when estimating TBW. The Imp™ SFB7 (SFB7) is based on similar BIS principles, while offering increased portability and a greater range of frequencies when compared to older devices, such as the XiTRON 4000B (4000B). The purpose of this study was to examine the validity of the SFB7 for estimating total body water in healthy college-age men and women compared to the 4000B and deuterium oxide (D_2_O).

**Methods:**

Twenty-eight Caucasian men and women (14 men, 14 women; 24 ± 4 yrs; 174.6 ± 8.7 cm; 72.80 ± 17.58 kg) had their TBW estimated by the SFB7, the 4000B, and D_2_O.

**Results:**

Both BIS devices produced similar standard error of estimate (*SEE*) and *r *values (SFB7, *SEE *= 2.12L, *r *= 0.98; 4000B, *SEE *= 2.99L, *r *= 0.96) when compared to D_2_O, though a significant constant error (*CE*) was detected for the 4000B (2.26L, *p *≤ 0.025). The 4000B produced a larger total error (*TE*) and *CE *(*TE *= 3.81L, *CE *= 2.26L) when compared to the SFB7 (*TE *= 2.21L, *CE *= -0.09L). Additionally, the limits of agreement were larger for the 4000B (-3.88 to 8.39L) than the SFB7 (-4.50 to 4.31L). These results were consistent when sex was analyzed separately, though women produced lower *SEE *and *TE *values for both devices.

**Conclusion:**

The 4000B and SFB7 are valid BIS devices when compared to D_2_O to estimate TBW in college-age Caucasian men and women. Furthermore, the new SFB7 device displayed greater precision in comparison to the 4000B, which may decrease the error when estimating TBW on an individual basis.

## Background

Estimating total body water (TBW) has been widely used to increase the accuracy of body composition measurements [1]. Specifically, Wang et al. [1] found that, compared to a six-compartment model, the best methods for estimating body fat percentage included an estimation of TBW. However, considering the two-compartment model based on TBW measurements and appropriate hydration factors, the hydrometry method is accurate and precise to estimate fat-free mass [2]. Additionally, TBW estimations have been used to identify and monitor diseases and nutrition status [3-6]. Criterion isotope methods for estimating TBW, such as deuterium oxide, hydrogen, tritium, oxygen-18, and oxygen, are time-consuming and expensive and require cumbersome equipment and techniques. Bioimpedance spectroscopy (BIS) has been used as an alternative to these isotope methods due to its reduced administration time, ease of use, and lower cost [7-11]. BIS uses a range of frequencies encompassing both low and high ranges that allow electrical current to pass around and through each cell. This technique, explained elsewhere [12], has produced valid measurements of TBW when compared to a criterion method, such as deuterium oxide [7,8,10-12]. However, past investigations on the validity of BIS have predominantly focused on one specific BIS model (XiTRON 4000B), and most studies examining healthy adult populations are limited to this device [7-10]. Moreover, the use of BIS to estimate TBW in individuals, rather than groups, has not been recommended due to large individual errors [13]. However, the underlying cause of these individual errors is unclear. Recently, a new device (ImpediMed Limited, Imp™ SFB7) has become available that adds portability to the myriad of BIS benefits via an onboard computer. The Imp™ SFB7 (SFB7) uses 256 frequencies ranging from 4 to 1000 kHz, while the XiTRON 4000B (4000B) incorporates 50 frequencies ranging from 5 to 1000 kHz, although, the effect of greater frequency utilization is not known. To the best of our knowledge, no previous study has compared the 256 frequency SFB7 to any criterion TBW technique. Therefore, the purpose of this investigation was 1) to compare the new SFB7 device to deuterium oxide (D_2_O) for estimating TBW, and 2) to compare the TBW values attained from the 50 frequency 4000B and the 256 frequency SFB7. It was hypothesized that both BIS devices would produce valid measurements compared to D_2_O and that the SFB7 would reduce the error between D_2_O and BIS due to the increased number of frequencies used for the estimation of TBW.

## Methods

### Subjects

Twenty-eight Caucasian men and women (19–35 years, 24 ± 4) volunteered to participate in the study (Table 1). All measurements were performed on the same day following a 12-hour fast (*ad libitum *water intake was allowed). The subjects were also instructed to refrain from exercising for at least 12 hours prior to testing. Hydration status was analyzed using specific gravity via a refractometer (Model CLX-1, precision = 0.001 ± 0.001, VEE GEE Scientific, Inc. Kirkland, Washington), and all subjects produced specific gravity values < 1.030 (1.019 ± 0.008, mean ± SD) indicating sufficient hydration [7,14]. Height and weight were measured via a stadiometer and calibrated physician's scale to the nearest 0.5 cm and 0.01 kg, respectively. The purpose of the study and a description of the testing protocol were explained to each subject. Additionally, the study was approved by The Institutional Review Board for Human Subjects, and written informed consent was obtained from each subject prior to testing.

**Table 1:** Subject descriptive characteristics and the validation of BIS for predicting total body water compared to deuterium oxide (*n *= 28, 14 men, 14 women)

											**Agreement (L)**			
											
	**Weight (kg) **$\bar{x}$**± SD**	**Height (cm) **$\bar{x}$**± SD**	**Method**	$\bar{x}$**± SD (L)**	**Slope**	**Intercept**	***r***	***r*^2^**	***SEE *(L)**	***TE *(L)**	***CE*/Bias**	**Upper Limits**	**Lower Limits**	**Trend**
**All Subjects**	72.8 ± 17.58	174.6 ± 8.7	4000B	38.28 ± 10.71	0.897	6.19*	0.96	0.92	2.99	3.81	2.26*	8.39	-3.88	-0.70
			SFB7	40.63 ± 10.69	0.920	3.18	0.98	0.96	2.12	2.21	-0.09	4.31	-4.50	-1.32
			D_2_O	40.54 ± 10.05										
**Men**	87.10 ± 12.52	181.2 ± 6.4	4000B	47.51 ± 6.43	0.767	12.59	0.81	0.66	3.75	4.05	1.50	9.15	-6.15	-0.13
			SFB7	49.81 ± 6.29	0.879	5.22	0.91	0.83	2.70	2.73	-0.80	4.49	-6.11	-0.15
			D_2_O	49.01 ± 6.11										
**Women**	58.49 ± 6.67	168.1 ± 4.9	4000B	29.05 ± 3.68	1.014	2.62	0.88	0.77	2.05	3.56	3.01*	6.87	-0.85	0.55
			SFB7	31.44 ± 4.00	0.991	0.92	0.94	0.88	1.50	1.52	0.62	3.45	-2.20	0.43
			D_2_O	32.06 ± 3.68										

* Represents significance at (*p *≤ 0.025), SFB7 = Imp™ SFB7, 4000B = XiTRON 4000B, *CE*/Bias = constant (mean) error,*TE *= total error, *SEE *= standard error of estimate, *r *= Pearson product-moment correlation coefficient, Limits = 95% limits of agreement (*CE *± 1.96 SD of residual scores (predicted – actual)), Trend = relationship between the difference of D_2_O and BIS method (SFB7, 4000B) and the mean of both methods.

### Bioimpedance spectroscopy

TBW was measured by bioimpedance spectroscopy using the SFB7 (ImpediMed Limited, Queensland, Australia) and 4000B (XiTRON technologies, San Diego, CA) devices following the manufacturer recommended procedures. Briefly, after resting in a supine position for 5 to 10 minutes, total body water estimates were taken while the subjects lay supine on a table with their arms ≥ 30 degrees away from their torso with their legs separated. The average of two trials was used to represent the subject's TBW. Prior to analysis, each subject's height, weight, and sex were entered into the SFB7 and 4000B devices, with the inclusion of age for the SFB7. From the two trials that represented each of the 28 subject's TBW, consecutive test-retest reliability for the SFB7 and 4000B produced standard error of measurements (*SEM*) of 0.04 liters and 0.32 liters, respectively. These methods are similar to previously published BIS research [8-10,12]. Both BIS devices calculated TBW from the equations derived by Hanai [15]. These equations calculate TBW by combining extra-cellular water and intra-cellular water with the addition of coefficients and complex impedance plots [7,12,15].

### Deuterium oxide

A D_2_O tracer was used as the criterion method to estimate TBW. Prior to D_2_O ingestion, urine samples were collected from all subjects. Subjects were instructed to void their bladder as much as possible. After voiding the bladder completely, subjects ingested ≈ 11 grams of D_2_O along with a 100 ml rinse of deionized water. The exact amount of deuterium oxide ingested for each subject was recorded. After a 4-hour equilibration period restricting defecation, urination, and food and water ingestion, subjects were instructed to provide a post-urine sample. Within 30 minutes of collection, all urine samples were pipetted into cryogenic vials and stored at -80°C for later analysis [10,14]. At an independent laboratory (Metabolic Solutions, Inc., Nashua, NH.), the urine-diluted D_2_O was analyzed in triplicate using an isotope-ratio mass spectrometer, and the isotope abundances in the urine were calculated following the method of Wong et al. [16]. TBW was then calculated from the dilution of isotopic water and corrected for the exchange of deuterium with nonaqueous tissue [17].

### Data analysis

Validity of TBW estimates (SFB7 and 4000B) was based on an evaluation of predicted values versus the criterion (actual value) D_2_O by calculating the constant error (*CE *= actual TBW (D_2_O) – predicted TBW (BIS)), *r *value, standard error of estimate (*SEE *= SD $\sqrt{1-{\text{r}}^{2}}$), and total error (*TE *= $\sqrt{\sum {\left[\text{predicted}-\text{actual}\right]}^{2}/\text{n}}$) [18,19]. The mean difference (*CE*) between the predicted (SFB7 and 4000B) and actual (D_2_O) TBW values was analyzed using dependent *t*-tests with the Bonferroni alpha adjustment (*p *≤ 0.025) [20]. Additionally, the method of Bland and Altman [21] was used to identify the 95% limits of agreement between the criterion and predicted TBW values.

## Results

**Figure 1:**
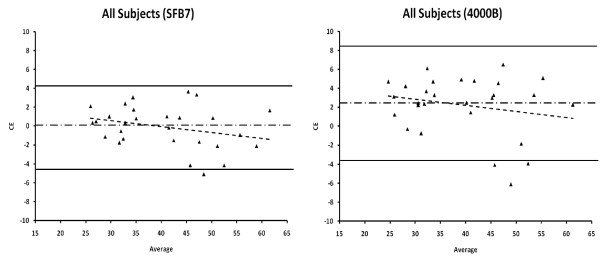
Bland and Altman plots of all 28 subjects comparing total body water (TBW) estimations by the SFB7 (Imp™ SFB7) and 4000B (XiTRON 4000B) to deuterium oxide (D_2_O). CE = constant error [CE = TBW D_2_O - TBW BIS (SFB7, 4000B)]; Average = [TBW D_2_O + TBW BIS (SFB7, 4000B)]/2. The solid lines represent the upper and lower limits of agreement (± 1.96 SD). The dotted/dashed line represents the constant error or mean bias. The dashed regression line represents the trend between the differences of methods and the mean of both methods.

The criterion D_2_O TBW values are presented in Table 1 along with the results of the validation analyses. Regression analysis for the 4000B resulted in a significantly different y-intercept (*p *≤ 0.025) for all subjects (y-intercept = 6.19) compared to a y-intercept of zero; however, when men and women where compared separately the y-intercepts were not significantly different than zero. Additionally, all slope values were not significantly different than 1.0 (*p *> 0.05). Constant error values ranged from 3.01L (4000B, women) to -0.09L (SFB7, all subjects) with significant *CE *differences (*p *≤ 0.025) detected for the 4000B in all the subjects and for only the women. The lowest validity coefficient was 0.81 (4000B, men), and the highest was 0.98 (SFB7, all subjects), while the *SEE *values ranged from 1.5 L (SFB7, women) to 3.75 L (4000B, men). *TE *values from the 4000B (*TE *≥ 3.56 L) were larger than the TE values produced by the SFB7 (*TE *≤ 2.73 L). The 95% limits of agreement were the largest for the 4000B, while the SFB7 produced smaller limits of agreement (Figure 1).

## Discussion

In accordance with our hypothesis, both BIS devices produced valid estimations of TBW compared to D_2_O in college-age Caucasian men and women. The results of the current study suggest that the 4000B and SFB7 are valid laboratory methods when compared to D_2_O to estimate TBW in this population. However, the use of the newer SFB7 reduced individual TBW errors and, therefore, is recommended over the 4000B for use in small groups or individuals.

In agreement with previous literature, the 4000B and SFB7 produced an *r *value > 0.91 (4000B, *r *= 0.96; SFB7, *r *= 0.98) and a low *SEE *(4000B, *SEE *= 2.99 L; SFB7, *SEE *= 2.12 L) [8-10]. Specifically, in healthy college-age men and women, van Marken Lichtenbelt et al. [10] produced an *r *value of 0.98 using the 4000B, which is similar to the current findings for both devices (4000B, *r *= 0.96; SFB7, *r *= 0.98). Van Loan et al. [11] found a *SEE *value of 2.59 L in slightly older men and women (mean age = 29.9 years) using the XiTRON 4000, which is similar to the *SEE *values in the current investigation (4000B, *SEE *= 2.99 L; SFB7, *SEE *= 2.12 L). To the best of our knowledge, there is no extant literature involving the validity of BIS to estimate TBW in healthy women alone, and only one study has looked at the validity of the 4000B versus D_2_O in men alone. Armstrong et al. [7] found slightly better agreement between the 4000B and D_2_O (*SEE *= 2.23 L, *r *= 0.96) in college-age men compared to the current findings with the 4000B (*SEE *= 3.75 L, *r *= 0.81). However, the SFB7 produced comparable results (*SEE *= 2.70 L, *r *= 0.91) to those found by Armstrong et al. [7].

Overall, the *SEE *and *r *values from both the 4000B and SFB7 agree with past BIS research in healthy adult men and women [7-11]. However, the largest discrepancies between the current investigation and past literature are the *CE *values. The TBW *CE *values for all subjects (*CE *= 2.26 L) and the women (*CE *= 3.01 L) were significantly lower (*p *< 0.025) than the D_2_O TBW values, which is inconsistent with past findings [7-11]. The SFB7 produced no significant differences in TBW for all subjects, men, and women compared to the D_2_O TBW values. These *CE *differences cannot be seen in the *SEE *values; however, the effect of the *CE *on the *SEE *can be seen in the *TE *value, which accounts for errors associated with both the *CE *and *SEE *[22].

In all of the subjects, men, and women, the *TE *values for the 4000B were greater than the *TE *values from the SFB7 (Table 1), indicating that the SFB7 is more accurate for predicting TBW. Nonetheless, the significantly different *CE *values indicate a systematic error in the device. This systematic error may be due to the age of the device (11 years) or the coefficients used to estimate TBW; however, this error requires further research and is not the focus of this investigation. Ultimately, when comparing the 4000B and the SFB7, regardless of the systematic underestimation, the SFB7 produced lower *SEE *values and greater *r *values for all groups (Table 1).

**Figure 2:**
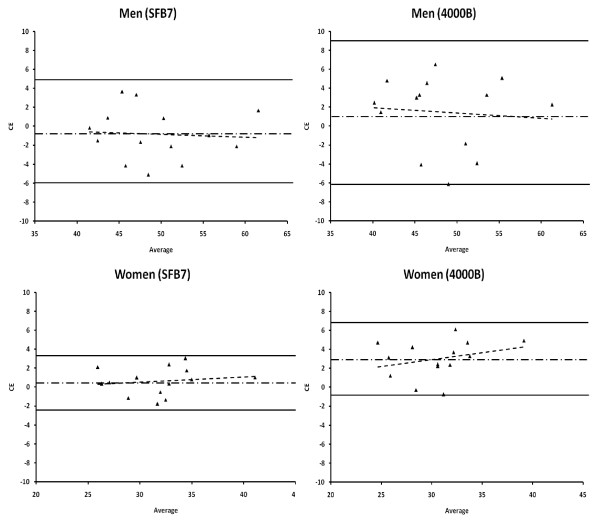
Bland and Altman plots of men and women comparing total body water (TBW) estimations by the SFB7 (Imp™ SFB7) and 4000B (XiTRON 4000B) to deuterium oxide (D_2_O). CE = constant error [CE = TBW D_2_O - TBW BIS (SFB7, 4000B)]; Average = [TBW D_2_O + TBW BIS (SFB7, 4000B)]/2. The solid lines represent the upper and lower limits of agreement (± 1.96 SD). The dotted/dashed line represents the constant error or mean bias. The dashed regression line represents the trend between the differences of methods and the mean of both methods.

Additionally, individual subject results can be compared by calculating the limits of agreement (Table 1) [21]. These limits indicate that for all subjects (Figure 1) and for men and women (Figure 2), the SFB7 is more accurate than the 4000B. Moreover, the 4000B may over-predict TBW by as much as 3.88 L and under-predict by as much as 8.39 L in all subjects, while the SFB7 may over-predict TBW by as much as 4.50 L and under-predict by as much as 4.31 L in all subjects. For both devices these limits improved for the women and did not improve for men. The variations in the limits of agreement between sexes are most likely due to the coefficients used to estimate TBW and the larger TBW values for men (49.01 ± 6.11) compared to the women (32.06 ± 3.68). Additionally, there was a slight non-significant trend (*p *> 0.05) for both devices to overestimate TBW as TBW increased in all subjects and men (Table 1), while there was a slight non-significant trend (*p *> 0.05) for both devices to underestimate TBW as TBW increased in women.

Limitations of this study include the use of an outdated BIS device as a comparison for a new BIS device. However, it can be assumed that many of these 4000B devices are still in use today. Based on the current findings, future research should evaluate the validity of BIS devices throughout their span of use in order to determine if a device can remain valid over time. Although we calibrated the 4000B prior to each test, we cannot determine the specific cause of the significant *CE *values. Additionally, we did not directly test the hypothesis that "the SFB7 would reduce the error between D_2_O and BIS due to the increased number of frequencies used for the estimation of TBW"; however, it was determined that the increased number of frequencies utilized in the SFB7 device may have contributed to the more accurate estimations of TBW. Nonetheless, more research is required to identify if the number of frequencies utilized in a BIS device improves TBW measurements. Future research should compare TBW measurements calculated from complex impedence plots generated using various numbers of frequencies by the same BIS device in order to identify if the number of frequencies utilized actually improves TBW measurements.

In conclusion, the BIS method for estimating TBW in healthy individuals requires additional research in order to further reduce individual errors. While the new SFB7 device improves upon the older 4000B, there is still a small margin of disagreement between BIS and D_2_O TBW values. However, both the 4000B and SFB7 are apparently valid, non-invasive, portable devices for estimating TBW in college-age Caucasian men and women, with greater accuracy in the women. Future research should include a larger sample size and categorize healthy populations based on TBW in order to generate more accurate coefficients.

## Competing interests

The author(s) declare that they have no competing interests.

## Authors' contributions

JM, ST, MR, VD, CK, MB, JC, JS participated in the study design and helped draft the manuscript while JM, MR, VD, and CK, aided in data collection. Additionally, all authors read and approved the final manuscript.
